# Blue Wavelength of Scanning Laser Ophthalmoscope Potentially Detects Arteriosclerotic Lesions in Diabetic Retinopathy

**DOI:** 10.3390/diagnostics14131411

**Published:** 2024-07-02

**Authors:** Shintaro Horie, Yudai Suzuki, Takeshi Yoshida, Kyoko Ohno-Matsui

**Affiliations:** 1 Department of Ophthalmology and Visual Science, Tokyo Medical and Dental University, Tokyo 113-8510, Japan; 2 Department of Ophthalmology, Tokyo Metropolitan Tama Medical Center, Tokyo 113-8510, Japan; 3 Department of Advanced Ophthalmic Imaging, Tokyo Medical and Dental University, Tokyo 113-8519, Japan

**Keywords:** retinal imaging, scanning laser ophthalmoscopy, arteriosclerosis

## Abstract

(1) Background: The fundus examination is one of the best and popular methods in the assessment of vascular status in the human body. Direct viewing of retinal vessels by ophthalmoscopy has been utilized in judging hypertensive change or arteriosclerosis. Recently, fundus imaging with the non-mydriatic scanning laser ophthalmoscope (SLO) has been widely used in ophthalmological clinics since it has multimodal functions for optical coherence tomography or angiography with contrast agent dye. The purpose of this study was to examine the utility in detecting arteriosclerosis of retinal vessels in SLO images; (2) Methods: Both color and blue standard field SLO images of eyes with diabetic retinopathy (DR) were examined retrospectively. Retinal arteriosclerosis in color SLO images was graded according to the Scheie classification. Additionally, characteristics of retinal arterioles in blue SLO images were identified and examined for their relevance to arteriosclerosis grades, stages of DR or general complications; (3) Results: Relative to color fundus images, blue SLO images showed distinct hyper-reflective retinal arterioles against a monotone background. Irregularities of retinal arterioles identified in blue SLO images were frequently observed in the eyes of patients with severe arteriosclerosis (Grade 3: 79.0% and Grade 4: 81.8%). Furthermore, the findings on arterioles were more frequently associated with the eyes of DR patients with renal dysfunction (*p* < 0.05); (4) Conclusions: While color SLO images are equally as useful in assessing retinal arteriosclerosis as photography or ophthalmoscopy, the corresponding blue SLO images show arteriosclerotic lesions with high contrast in a monotone background. Retinal arteriosclerosis in eyes of advanced grades or advanced DR frequently show irregularities of retinal arterioles in the blue images. The findings of low, uneven, or discontinuous attenuation were easier to find in blue than in color SLO images. Consequently, blue SLO images can show pathological micro-sclerosis in retinal arterioles and are potentially one of the safe and practical methods for the vascular assessment of diabetic patients.

## 1. Introduction

Since retinal arterioles are unique vessels that can be directly viewed during whole-body examinations, fundus photography or ophthalmoscopic examination has long been a standard method in detecting arteriosclerosis [1,2,3]. The Scheie classification of hypertension and arteriosclerosis [4] has been a popular grading method in clinical assessments by both ophthalmologists and physicians [5,6]. The findings indicating arteriosclerosis in retinal vessels were graded into four scales corresponding to the appearance of vessel walls. The earliest stage is defined as a mild reflex change in vessel walls, while entirely white vessels with hyper-reflection represent the most severe stage [4]. Recently, automated retinal image analysis software has reached the clinical stage and has the potential to substitute for conventional photo-based screening by ophthalmologists or physicians [7]. Previous studies reported reliable sensitivity and specificity of an autonomous-AI diagnostic system in diagnosing any stage of DR [8,9,10,11,12,13,14].

While digitized data other than film are a standard method in recordings today, most commercially available fundus cameras still adopt the optical system with visible light. However, scanning laser ophthalmoscopy (SLO) is becoming more popular in ophthalmology clinics since the instrument has advanced usefulness in capturing wide-field images in a single shot without mydriasis [15,16,17,18,19,20]. Recently, multimodal assessments with optical coherence tomography (OCT), microperimetry or fluorescein/indocyanine green angiography together with color photography have been recommended in retinal clinics for optimum assessment [21]. Some of these functions are often combined with SLO systems, enabling complete assessments of retinal diseases with a single instrument. 

Today, commercially available SLO instruments utilizes red and green laser emissions with or without blue wavelengths. The color fundus image obtained by SLO is not in the real colors of natural visible light, but synthesized from two or three monowavelength images. Usually, the synthesized color image is accepted as a “real color” image, while monotone single-color images of each wavelength are not clinically utilized yet. Previously, we reported that blue monowavelength SLO images show retinal non-perfusion areas in diabetic retinopathy (DR). Fluorescein angiography (FA), which is a standard method used in detecting non-perfusion areas, requires dye infusion, which presents a risk in patients with renal failure. Furthermore, potential anaphylactic reactions to the agent are also a risk. Thus, utilizing blue SLO images has certain benefits in the context of identifying disease progression in DR patients with minimal hazardous risk [19].

Like the wide-field images of SLO, standard field color images of SLO consist of multiple monowavelength images. The monotone image is simultaneously available with the color one. Little is known yet about the characteristics or utility of monowavelength SLO retinal images. Although these images appear less informative, it is assumed they have some advantage by offering information with relatively high contrast compared to color images. 

Here, we examine and report characteristic findings of retinal arterioles observed in blue SLO images of eyes of patients with diabetes, which may have clinical importance in vascular assessment. While retinal arteriosclerosis grading or morphological change in arterioles has not received as much attention as micro-capillary dropout or venous change in DR, that does not imply arteriole pathology is of less clinical importance. This practical method without hazardous risk offers potential benefits for all patients with or without DR.

## 2. Materials and Methods

### 2.1. Study Design and Patients

This study was a retrospective observational case series. We examined the medical records and SLO images of patients with diabetic retinopathy (DR) who had been examined in the outpatient clinic of the Department of Ophthalmology at Tokyo Medical and Dental University Hospital. All the subjects had undergone multicolor standard field SLO imaging with Mirante ^®^ (NIDEK, Gamagori, Aichi, Japan) between January 2021 and February 2024. The patients with DR were classified as PDR, moderate to severe PDR, or less than mild NPDR according to a classification by the global diabetic retinopathy project group [22]. Patients under 20 years of age and those whose SLO images were not clear due to media opacities of severe cataract or vitreous hemorrhage, etc., were excluded. History of general complications such as hypertension or renal dysfunction of chronic kidney disease or hemodialysis was retrospectively checked through medical records. The Ethics Committee of Tokyo Medical and Dental University approved the procedures and waived the requirement for informed consent from all subjects in this data-based retrospective observational study. The research procedures conformed to the tenets of the Declaration of Helsinki.

### 2.2. Multicolor Scanning Laser Ophthalmoscope (SLO) 

All patients were examined by standard field multicolor SLO fundus imaging by Mirante ^®^ (NIDEK, Gamagori, Aichi, Japan). This optical instrument utilizes blue (488 nm), green (532 nm) and red (670 nm) emission wavelengths, generating synthesized color images with these non-color single-wavelength images. In this study, both the blue and synthesized color SLO images were compared and analyzed. Initially, the identification and grading of the images were performed by two of the authors (SH and YS) independently according to the Scheie classification of arteriosclerosis: Grade 1, there is broadening of the light reflex from the arteriole with minimal or no arteriovenous compression; Grade 2, light reflex changes and crossing changes are more prominent; Grade 3, the arterioles have a copper wire appearance, and there is more arteriovenous compression; and Grade 4, the arterioles have a silver appearance, and arteriovenous crossing changes are most severe [4]. Irregularities of retinal arterioles were included with irregular deformity, low attenuation, or discontinuity of white vessels identified by blue SLO images. The graders were masked to the background information on the patients of each image. In cases of discrepancies in initial grading by the two graders, the final grade of each image was determined by consensus of both graders.

### 2.3. Statistical Analyses

Fisher’s exact tests were used to determine the significance of the relations between blue SLO findings (irregularity of arterioles) and history or lab data of general complications (hypertension or renal dysfunction) from medical records. A *p*-value of <0.05 was considered statistically significant. The odds ratio was also calculated. The cases analyzed were not consecutive, and the sample size was not optimized. Statistical analyses were performed using GraphPad Prism (GraphPad Software. Inc. Ver. 6.0).

## 3. Results

### 3.1. Demographics of Patients and Retinal Arteriosclerosis Grading of DR Patients

The medical records of 219 eyes of 116 DM patients (77 men and 39 women) were reviewed; 99 eyes of 60 patients had PDR, 76 eyes of 45 patients had severe NPDR, and 44 eyes of 25 patients had less than moderate NPDR. Fourteen patients had signs of a different stage in one eye from that of the other. Of the 219 eyes, 157 had received pan-retinal retinal photocoagulation: 105 eyes with PDR, 52 eyes with severe NPDR, and none with less than moderate NPDR. Additionally, 95 of the 219 eyes had received anti-VEGF therapies, and 54 of the PDR eyes had undergone pars plana vitrectomy.

Standard-field color SLO images were available for all 219 eyes of the 116 DM patients. We examined 99 eyes with PDR, 76 eyes with severe NPDR and 32 eyes with less than moderate NPDR, and arteriosclerosis findings in each image were graded from 0 (none) to 4 (highest) according to the Scheie classification. Among all eyes examined, 78 were graded as Grade 0 or Grade 1, 57 were graded as Grade 2, 62 were graded as Grade 3, and 22 were graded as Grade 4. The eyes graded as Grade 4 included only one eye of severe NPDR and none of less-than-moderate NPDR (Table 1). 

### 3.2. Irregularities of Retinal Arterioles Were Frequently Identified in Eyes with Severe Arteriosclerosis or Advanced Stages of DR Eyes in Blue SLO Images

**Figure 1:**
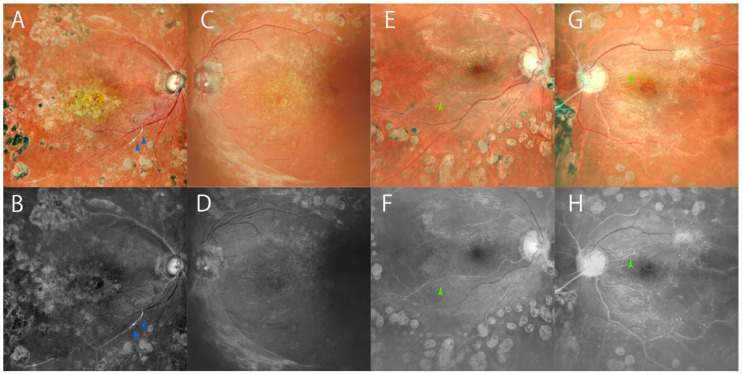
Color and blue SLO images show irregular hyper-reflective appearance of retinal arterioles in DR eyes. (**A**) Color SLO image of right eye of a 72-year-old woman with PDR. (**B**) Blue SLO image of (**A**) shows white arterioles indicating severe arteriosclerosis (Grade 3). Irregularities (blue arrowheads) are observed. (**C**) The color image of fellow eye of (**A**) (Grade 3). (**D**) Corresponding blue SLO image of C showing uneven white arterioles. (**E**) Color SLO image of right eye (Grade 3) of a 70-year-old woman with low-attenuated irregular arteriole (green arrowhead). (**F**) Corresponding blue SLO image of (**E**). The arteriole (green arrowhead) is more easily identified in the blue SLO image. (**G**) The color image of fellow eye of (**E**) (Grade 4). (**H**) Corresponding blue SLO image of (**G**). The arteriole shows uneven irregular hyper-reflection.

Relative to color fundus images, blue SLO images of DR patient eyes displayed distinct hyper-reflective whiteness of retinal arterioles against a monotone background. In many cases, low-attenuated white arterioles are hard to find in color images, while they are easier to be identified in blue ones (Figure 1). In considerable numbers of blue and color SLO images, arterioles showed an uneven appearance of hyper-reflective vessels with irregular shapes, low attenuations, or discontinuity (Figure 1), which might reflect exact focal arteriosclerosis. Based on this finding, the incidence of irregular retinal arterioles found in blue SLO images was examined. 

Hyper-reflective vessels with irregular appearance on blue SLO images were identified in 50 of 99 PDR eyes (50.5%), 38 of 76 severe NPDR eyes (50.0%), 13 of 44 less-than-moderate NPDR eyes (29.5%) and 101 of 219 DM eyes (46.1%). According to the arteriosclerosis grades in DM eyes, 18 of 22 Grade 4 eyes (81.8%), 49 of 62 Grade 3 eyes (79.0%), 26 of 57 Grade 2 eyes (45.6%), and 8 of 78 Grade 0 or 1 eyes (16.0%) showed irregularity of retinal arterioles (Table 2). Consequently, this significant finding in blue SLO images was frequently observed both in the eyes with severe arteriosclerosis (Grade 4: 81.8%) and the eyes with more than severe NPDR (Table 2).

### 3.3. Irregular Hyper-Reflective Retinal Arterioles Observed in Blue SLO Images Is Possibly Relevant to Renal Dysfunction in Patients with DR

Hyper-reflection with irregularity in blue SLO image was found in all cases with Grade 4 arteriosclerosis in either eye (100.0%), 31 of 37 cases with Grade 3 eyes (83.8%), 14 of 27 cases with Grade 2 eyes (51.9%), and 8 of 37 cases with Grade 1 or 0 eyes (21.6%), and in 64 of 116 DM patients (55.1%) (Table 3). Consequently, the cases with severe arteriosclerosis (Grade 3 or 4) in either eye were likely to show the findings more frequently. Furthermore, the relevance between the incidence of the observation and general complications of hypertension or renal dysfunction was examined. In terms of hypertension, 41 of 64 cases with the irregular form of white arterioles in either eye (62.1%) had the complications, while 31 of 52 cases without the sign had them (60.0%) as well. The relevance was not statistically significant (Fisher’s exact test). However, 34 of 64 cases with the findings in either eye (53.1%) had renal dysfunction, while 17 of 52 cases without the sign also had it (32.7%), showing an odds ratio of 2.33 and statistical significance (*p* = 0.038) (Table 3).

## 4. Discussion

In this study, we reported a novel advantage of utilizing single-wavelength SLO images in retinal vascular assessment. The standard-field SLO images of blue wavelength in this study showed hyper-reflective retinal arterioles more clearly with a high-contrast monotone background than did the corresponding color images. 

The eyes with DR included in our study consisted of 99 eyes with PDR, 76 eyes with severe NPDR, and 44 eyes with less-than-moderate NPDR. Presumably, due to the relatively high proportion of severe DR eyes in this study, significant numbers of the eyes included here were graded as Grade 3 (62 eyes) or Grade 4 (22 eyes) arteriosclerosis. Accordingly, many cases of severe arteriosclerosis were included in this study.

In general, arteriosclerosis in retinal vessels is graded according to appearance and color change of vessel walls; most early-stage cases are defined as mild reflex change in vessel walls, while entirely white hyper-reflective vessels are the most severe stage (Grade 4) [4]. A color SLO image, although it is of synthesized colors processed by two (red and green) or three (additional blue) wavelengths, looks almost identical to a photograph obtained by a classical fundus camera [7,20]. Accordingly, the interpretation of a finding seen on a color SLO image is usually identical to that based on conventional photography, and we assumed the grading of retinal arteriosclerosis could be conducted in a way similar to that achieved with the classical method. However, hyper-reflections of retinal arterioles with white appearance, which indicated arteriosclerosis, were hard to find in some cases on color SLO image due to low contrast. This was more likely in low-attenuated or obstructive arterioles in relatively advanced stages. Conversely, we noticed that such indistinctive or subtle irregularities were easier to find on blue SLO images. Furthermore, we obtained more details on arteriole appearance with hyper-reflections on blue image. In this study, we found irregular patterns of hyper-reflective arterioles on many blue SLO images, which potentially showed precise arteriosclerotic lesions in vivo. In considerable numbers of blue SLO images, retinal arterioles presented hyper-reflective vessels with irregular shapes of caliber difference, low attenuations, or discontinuity (Figure 1), potentially reflecting exact focal arteriosclerosis. The hyper-reflective arterioles observed on those images showed intermittent white lines, and they were frequently identified in 1st or 2nd retinal arterioles. Discontinuities or interruptions of arterioles are more likely to be found in thinner arterioles assumed to be pre-capillary arterioles. As summarized in Table 2, this finding was frequently observed both in eyes with severe arteriosclerosis (Grade 4: 81.8%) and eyes with more than severe NPDR. Because the arteriosclerosis lesions were more prominent and variable in these conditioned eyes, proportionally more irregularities of vessels were likely to be found in those eyes. Since the hyper-reflection was not uniform, as presented in Figure 1A (arrowhead), the hyper-reflection potentially indicated arteriosclerotic calcification of the vessel wall. 

**Figure 2:**
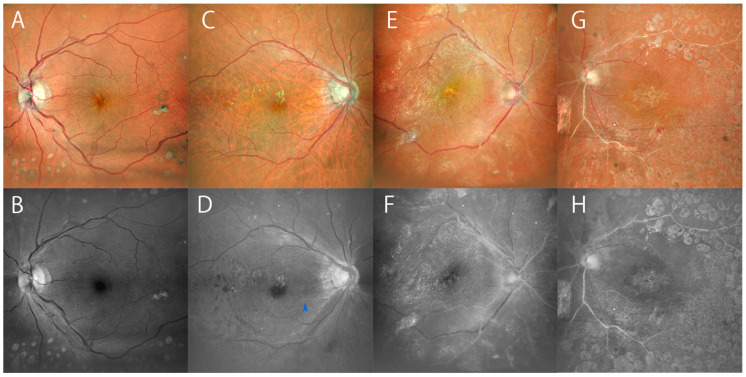
Blue SLO image shows arteriosclerosis with white appearance of eyes with DR. (**A**) Color SLO image of left eye (Grade 1) of a 55-year-old woman with PDR without renal dysfunction but hypertension. (**B**) Blue SLO image of A with mild hyper-reflective arterioles. (**C**) Color SLO image of right eye (Grade 2) of a 71-year-old man with moderate NPDR, chronic kidney disease (CKD) and hypertension. (**D**) Corresponding blue SLO image of (**C**) showing moderate hyperreflective arterioles with irregularity (blue arrowhead). (**E**) Color SLO image of right eye (Grade 3) of a 56-year-old man with PDR, CKD and hypertension. (**F**) Corresponding blue SLO image of (**E**). Severe hyper-reflective arterioles are easier to identify in the blue image. (**G**) Color SLO image of left eye (Grade 4) of a 50-year-old man with PDR, renal failure (hemodialysis) and hypertension. (**H**) Corresponding blue SLO image of (**G**). The distinct hyper-reflective arterioles also show irregularities.

Additionally, we examined the rate of findings in blue SLO images of patients with a history of general complications commonly accompanying DM. Among DM patients with a history of hypertension, irregularities of retinal arterioles in blue SLO images were frequently found in eyes with advanced arteriosclerosis. However, there were no significant differences in irregular findings in all patients with hypertension (72 cases). Conversely, among all DM patients with renal dysfunction (51 cases), the percentage of those with irregularities found in the fundus of either eye on SLO images was significantly higher (*p* = 0.038). This suggests that lesions of retinal arteriosclerosis are possibly related to the history of renal dysfunction in DM patients. As shown in Figure 2G,H, the SLO image of the eye of a patient undergoing hemodialysis shows severe arteriosclerosis with an entirely white hyper-reflective appearance. Simultaneously, the vessels show irregularities of arterioles as well. This result seems logical given the evidence that patients with chronic renal failure [23] or hemodyalysis accompanied by vascular calcification face a major risk of developing arteriosclerosis [24]. 

In our previous study, we examined the utility of single-wavelength SLO imaging for DR patients in detecting retinal non-perfusion areas (NPAs) other than by FA [19]. Since a single-wavelength monotone image is simultaneously available with the color one, little is known about its characteristics, and it has not been practically utilized in clinical settings. In the study, a significant concordance of the incidence of hyporeflective areas in the blue SLO and NPAs in the FA images was confirmed. Furthermore, the locations of hyporeflective areas in the blue SLO images were also found in the area of the NPAs identified in the FA images in patients with PDR and moderate-to-severe NPDR [19]. In this previous study, we also noticed that white vessels were often observed in hyporeflective area in blue SLO images. The vessels were assumed to be arteriole structures remaining in the NPAs. Inspired by this observation, we examined the characteristics of arterioles in blue SLO images and here reported the study of blue SLO imaging in DR.

As blue SLO images can be useful in detecting NPAs in severe DR, these previously overlooked images could be a similarly valuable tool for the assessment of most DR eyes. As shown above in Figure 1 and Figure 2, white low-attenuated arterioles are not easy to find in color images, due to their low contrast relative to the red fundus color, as well as through direct observation by ophthalmoscopy. This is potentially one of the reasons that arteriole changes have not had priority in the assessment of DR. However, no one can deny the importance of blood flow regulated by retinal arterioles. In comparisons between FA and blue SLO, although the black and white monotone images of FA look similar to those of blue SLO images, the white vessels in FA images indicate blood flow or pathologic staining of vessels, and are not identical to the hyper-reflective vessels in blue SLO images. Utilizing blue SLO images to find arteriosclerosis in DR eyes could help in assessing variable vascular lesions as well as capillary dropout or pathologic venous changes in DR eyes. 

In this study, the characteristic appearance of retinal arterioles identified in blue SLO images has importance since it is distinct in blue monotone images. The irregular patterns of hyper-reflective arterioles indicated focal deformity of vascular walls, low attenuation or obstruction. They were frequently found in the eyes of patients with high-grade arteriosclerosis (Grade 3 or Grade 4) and advanced stages of DR (PDR or severe NPDR). It is unsurprising that pathologic arterioles are frequently observed in these conditioned eyes [25,26,27]. Consequently, blue SLO images may be useful in identifying arteriole pathology and achieving a total vascular assessment of the DR eye. Additionally, statistical analysis indicates DM patients with renal dysfunction are likely to show irregular appearances of retinal arterioles in blue SLO images. It is known that patients undergoing hemodialysis have arteriosclerosis accompanied by severe calcifications [24,25]. In fact, consistent with this evidence, many eyes of DM patients who underwent hemodialysis in this study showed severe arteriosclerosis (Grade 3 or 4) as shown in Figure 2G,H. From these insights, it is speculated that the blue wavelength can detect calcifications with hyper-reflections, which are a major component of arteriosclerotic lesions.

The application of OCT angiographic (OCTA) findings as novel biomarkers of arteriosclerosis was also reported recently [28]. Compared to OCT angiography, which is one of the most recent technologies now widely used in DR clinics [28,29,30,31], blue SLO imaging may offer additional advantages in the assessment of retinal vasculature. They are both non-invasive methods that do not use dye infusion, and hence, relatively safe. SLO images can show vessels with the whole retinal image, while OCTA is almost specific in showing blood flow. Blue SLO images may detect structural components of the vessels walls, presumably arteriosclerosis plaques in particular.

This study has several limitations. It was not a consecutive case series, and the number of cases was not optimized for the statistics used. The study was conducted at a single tertiary eye center; thus, the results may not be applied to the general DR population. In the grading of images, agreement on the results by both graders was not analyzed in this study. General complications of renal function or hypertension status such as the grade or affected period might have varied in each patient. This probably affected the results of statistical analysis regarding the correlation of SLO findings with general histories of the patients in this study.

In summary, we suggest utilizing blue SLO images, which are acquired simultaneously with the color image, because the blue image shows advanced arteriosclerosis clearly with high contrast on a monotone background. This method is particularly worthwhile for finding irregular, low-attenuated or obstructive arterioles in eyes with advanced arteriosclerosis or severe-stage DR. While medical care for arteriosclerosis itself should be considered by the physicians, the ophthalmologist can inform them of valuable information. Furthermore, blue SLO images probably have similar usefulness in the assessment of eyes without DR, and are widely applicable for retinal vascular assessment.

## 5. Conclusions

This study highlights the practical usefulness of blue SLO images in the assessment of retinal arteriosclerosis. Blue SLO images of eyes with DR displayed more-distinct white hyper-reflective retinal arterioles against a monotone background compared to color fundus images. While low-attenuated white arterioles were hard to find in color images, they were easier to be identified in blue ones. In addition, considerable numbers of arterioles in blue images showed an uneven appearance of hyper-reflective vessels with irregular shapes or discontinuities, as well as low attenuation, presumably reflecting focal arteriosclerotic lesions. These characteristic findings were well observed both in advanced stages of arteriosclerosis and DR graded by color images. Furthermore, such arteriosclerosis with irregular findings in blue SLO images potentially correlates with the renal dysfunction of the DM patients. From these results, we suggest that ophthalmologists utilize blue SLO images as well as color fundus images to find arteriosclerotic lesions easily and precisely without requiring additional examinations of patients.

## Figures and Tables

**Table 1:** Retinal arteriosclerosis grade of DM patients graded by standard field color SLO image.

Arteriosclerosis Grade	DM Total Eyes	PDR	Severe NPDR	<Moderate NPDR
<Grade 1	78	20	30	28
Grade 2	57	22	22	13
Grade 3	62	36	23	3
Grade 4	22	21	1	0

DM = diabetes mellitus; PDR = proliferative diabetic retinopathy; NPDR = nonproliferative diabetic retinopathy.

**Table 2:** The incidence of irregularity of white retinal arterioles observed in blue SLO images of eyes with diabetic retinopathy.

Arteriosclerosis Grade	DM Eyes Irregularity(+)/Total	PDR Eyes	Severe NPDR Eyes	<Moderate NPDR Eyes
Total (Grade0–4)	101/219 (46.1%)	50/99 (50.5%)	38/76 (50.0%)	13/44 (29.5%)
<Grade 1	8/78 (16.0%)	3/20	3/30	2/28
Grade 2	26/57 (45.6%)	4/22	13/22	9/13
Grade 3	49/62 (79.0%)	26/36	21/23	2/3
Grade 4	18/22 (81.8%)	17/21	1/1	0/0

DM = diabetes mellitus; PDR = proliferative diabetic retinopathy; NPDR = nonproliferative diabetic retinopathy.

**Table 3:** General complications with or without irregularity of retinal arterioles in either eye of DM patients.

	Total (Arteriosclerosis Grade 0–4)		<Grade1		Grade 2		Grade 3		Grade 4	
Irregularity	(+)	(−)	(+)	(−)	(+)	(−)	(+)	(−)	(+)	(−)
Numbers of patients	64	52	4	33	14	13	31	6	15	0
Hypertension	41 (62.1%)	31 (60.0%)	2	17	5	10	22	4	12	0
Renal dysfunction *	34 (53.1%)	17 (32.7%)	1	5	4	8	18	4	11	0

DM = diabetes mellitus; PDR = proliferative diabetic retinopathy; NPDR = nonproliferative diabetic retinopathy; * *p* = 0.038 (Fisher’s exact test).

## Data Availability

The data presented in this study are available on request from the corresponding author. The data are not publicly available due to the intellectual property rights.
